# Carbon–nitrogen interaction controls postbiotic short-chain fatty acid spectrum in a bacterial–yeast consortium: a central composite design approach

**DOI:** 10.14202/vetworld.2026.1027-1042

**Published:** 2026-03-15

**Authors:** Yetti Marlida, Lili Anggraini, Tan Joo Shun, Syofyan Syofyan, Rima Dwitaviani, Laily Rinda Ardani, Thelsa Anggun Bagaskaraell

**Affiliations:** 1 Department of Animal Nutrition, Faculty of Animal Science, Universitas Andalas, Limau Manis Campus, Padang, West Sumatra 25163, Indonesia; 2 Research Organization of Agriculture and Food, National Research and Innovation Agency, Bogor, 16911, Indonesia; 3 Department of Bioprocess Technology, School of Industrial Technology, Universiti Sains Malaysia, Gelugor, Pulau Pinang, Malaysia; 4 Department of Pharmaceutics, Faculty of Pharmacy, Universitas Andalas, Limau Manis Campus, Padang, West Sumatra 25163, Indonesia; 5 Department of Biology, Faculty of Mathematics and Natural Sciences, Universitas Andalas, West Sumatra 25163, Indonesia; 6 Doctoral Program, Faculty of Animal Science, Universitas Andalas, Padang, West Sumatra 25163, Indonesia

**Keywords:** bacterial–yeast consortium, central composite design, *in vitro* rumen fermentation, nitrogen source optimization, postbiotic production, response surface methodology, short-chain fatty acids, *Schleiferilactobacillus harbinensis*

## Abstract

**Background and Aim:**

Postbiotics, particularly short-chain fatty acids (SCFAs), play critical roles in gut health, immune modulation, and animal productivity. However, nutrient-driven metabolic regulation of SCFA production in mixed microbial systems under rumen-simulated conditions remains poorly understood. This study aimed to optimize SCFA production and to evaluate how carbon (C) and nitrogen (N) concentrations, and incubation time, interact to control metabolic outputs in a bacterial–yeast consortium during *in vitro* rumen fermentation.

**Materials and Methods:**

A co-culture of *Schleiferilactobacillus harbinensis* LH991 and *Pichia kudriavzevii B-5P* was incubated anaerobically with goat rumen fluid using a response surface methodology–central composite design. Three variables were tested: glucose (0.1–0.3 g/L), yeast extract (5–15 g/L), and incubation time (24–72 h). Individual SCFAs (acetate, propionate, butyrate, iso-butyrate, valerate, and iso-valerate) were quantified by gas chromatography, and quadratic polynomial models were used to determine optimal conditions and interaction effects.

**Results:**

Model adequacy was confirmed with R² values ranging from 0.82 to 0.94 and non-significant lack-of-fit tests (p > 0.05). Optimal acetate production occurred at moderate C (0.2 g/L), N (10 g/L), and 48 h incubation. In contrast, propionate, butyrate, iso-butyrate, and iso-valerate production were maximized under low C (0.1 g/L), high N (15 g/L), and extended incubation (72 h). Valerate production showed dual optima depending on incubation duration and substrate balance. Response surface plots demonstrated clear nutrient-dependent metabolic shifts, indicating that N enrichment combined with C limitation redirected metabolic flux toward branched-chain and energy-dense SCFAs.

**Conclusion:**

This study demonstrates a previously unreported nutrient-dependent metabolic switching mechanism in a bacterial–yeast consortium under rumen-simulated conditions. Precise manipulation of C, N, and incubation time enables targeted modulation of SCFA profiles, providing a scalable strategy for cost-effective postbiotic production. These findings support the development of optimized microbial fermentation systems for animal nutrition, functional feeds, and industrial postbiotic applications.

## INTRODUCTION

Postbiotics, as defined by the International Scientific Association of Probiotics and Prebiotics, are preparations of inanimate microorganisms and their components that confer health benefits to the host [1]. These bioactive compounds include short-chain fatty acids (SCFAs), organic acids, peptides, enzymes, cell wall fragments, and immune-modulating metabolites produced during microbial fermentation or cell lysis [2]. Substances released during microbial cell lysis, such as teichoic acids, muropeptides, cell surface proteins, and polysaccharides, are also considered part of this spectrum. These compounds support intestinal health by enhancing the epithelial barrier, modulating immune responses, and reducing inflammation caused by pathogenic microbes [3].

Postbiotics exert a wide range of health-promoting effects, including immunomodulatory, anti-inflammatory, antioxidant, antihypertensive, antiproliferative, cholesterol-lowering, and anti-obesity properties [4]. Owing to their stability, safety, and ease of application, postbiotics have gained increasing attention as functional ingredients in animal feed, functional foods, and therapeutic applications [5]. SCFAs, particularly butyric acid, acetic acid, and propionic acid, as well as other bioactive molecules such as teichoic acid, indole, lipopolysaccharides, muramyl dipeptide, and lactospin, are among the most extensively studied postbiotic components [3]. These compounds play critical roles in maintaining gut health and systemic physiological balance.

The production efficiency and biological efficacy of postbiotics are highly dependent on incubation conditions, including the type and concentration of carbon (C) and nitrogen (N) sources, as well as incubation duration. Optimizing these parameters is essential for enhancing metabolite productivity and maximizing functional activities. Previous studies have demonstrated that targeted optimization strategies, such as response surface methodology, can significantly improve postbiotic production and highlight the potential of exopolysaccharides (EPS) with strong antioxidant and anti-inflammatory properties [6].

Rumen fermentation represents a unique anaerobic bioreactor system capable of producing diverse postbiotic metabolites through complex microbial interactions. Previous studies have shown that selected lactic acid bacteria (LAB) and yeast strains isolated from fermented products can enhance volatile fatty acid production while reducing methane emissions under *in vitro* rumen conditions [6, 7]. However, most postbiotic research has focused on single microbial cultures or non-rumen fermentation systems, with limited attention given to mixed bacterial–yeast co-culture systems.

In particular, systematic optimization studies evaluating how interactions among C source, N source, and incubation time influence the full spectrum of SCFAs in co-cultures of *Schleiferilactobacillus harbinensis* and *Pichia kudriavzevii* using rumen fluid-based incubation systems remain scarce. Previous studies have emphasized the potential of these microorganisms. For example, Susalam *et al*. [8] reported that *S. harbinensis* has significant probiotic potential in broiler chickens. Marlida *et al*. [9] demonstrated that *P. kudriavzevii* functions both as a probiotic and as a biosorbent for aflatoxin B1 in corn, while Anggraini *et al*. [10] showed that *P. kudriavzevii* can control aflatoxin contamination in broiler feeds. Furthermore, Marlida *et al*. [11] demonstrated that a 1:1 ratio of *S. harbinensis* to *P. kudriavzevii* with a 4% inoculum dose significantly increased SCFA production.

Although postbiotics and SCFAs have been widely investigated for their roles in gut health, immune regulation, and animal productivity, most studies have focused on single microbial strains or simplified fermentation systems. Previous investigations commonly evaluated LAB or yeast independently and frequently used artificial media rather than biologically relevant rumen-based environments. As a result, the metabolic interactions occurring in mixed bacterial–yeast consortia under rumen-simulated anaerobic conditions remain poorly understood. Furthermore, earlier research has typically examined fermentation factors in isolation, without systematically determining how C and N levels, and incubation time, interact to influence the complete SCFA profile.

For the consortium of *S. harbinensis* and *P. kudriavzevii*, earlier studies demonstrated probiotic potential, aflatoxin mitigation capacity, and enhanced SCFA production when combined. However, no comprehensive optimization study has quantified the joint regulation of metabolic outputs by nutrient balance and incubation duration in this co-culture. In particular, whether nutrient-driven metabolic switching selectively favors acetate, propionate, butyrate, or branched-chain acids has not been systematically evaluated using a multifactor statistical design. This knowledge gap hinders the development of efficient, scalable fermentation strategies for targeted postbiotic production in animal nutrition and functional feed systems. Therefore, an integrated evaluation using response surface methodology in a rumen-based co-culture system is necessary to clarify these interactions and determine optimal production conditions.

This study aimed to optimize postbiotic production by determining how C and N concentrations, and incubation time, interact to regulate SCFA synthesis in a co-culture of *S. harbinensis* and *P. kudriavzevii* under *in vitro* rumen fermentation conditions. Specifically, the objectives were to (i) evaluate the individual and combined effects of C level, N level, and incubation duration on the production of acetate, propionate, butyrate, iso-butyrate, valerate, and iso-valerate; (ii) identify optimal fermentation conditions for maximizing specific SCFAs using response surface modeling; and (iii) determine whether nutrient manipulation induces selective metabolic routing within the bacterial–yeast consortium.

By defining optimized fermentation parameters and clarifying nutrient-dependent metabolic responses, this study seeks to provide a scientific basis for designing scalable postbiotic production systems and improving microbial fermentation strategies for animal nutrition and functional feed applications.

## MATERIALS AND METHODS

### Ethical approval

Rumen fluid used for the *in vitro* incubation was collected from adult goats immediately after routine slaughter at a licensed local slaughterhouse. As the study did not involve any live-animal handling, experimental intervention, anesthesia, or euthanasia performed for research purposes, formal Animal Ethics Committee approval was not required under institutional policy for the use of abattoir by-products. Nevertheless, permission to collect rumen contents was obtained from the slaughterhouse management, and sampling was conducted only after slaughter as part of normal commercial operations, without altering animal handling or welfare procedures.

Rumen contents were collected within 15 min post-slaughter using an aseptic technique, transported in sealed sterile containers, and maintained under CO_2_ flushing to preserve anaerobiosis and minimize contamination. All personnel used appropriate personal protective equipment (laboratory coats, gloves, protective eyewear, and masks as required), and all procedures involving rumen fluid and microbial cultures were performed in accordance with institutional biosafety and hygiene protocols and under Biosafety Level 2 practices (e.g., controlled access, disinfected work surfaces, safe handling of biological materials, and appropriate waste segregation).

All biological waste (rumen residues, culture materials, and disposables) was decontaminated (e.g., autoclaving and/or approved chemical disinfection) before disposal according to institutional and national biohazard waste management regulations. No human participants were involved; therefore, informed consent was not applicable.

### Study period and location

This study was conducted from April to December 2024 at the Faculty of Animal Science, Feed Industry Technology Laboratory, Ruminant Nutrition Laboratory, and Non-Ruminant Nutrition Laboratory, Universitas Andalas, Indonesia. SCFA analysis was performed at the Indonesian Research Institute for Animal Production (Balitnak), Bogor, Indonesia.

### Microbial strains and preparation of inoculum

The probiotic strains used in this study were *S. harbinensis* LH991 and *P. kudriavzevii* B-5P, obtained from the culture collection of the Feed Industry Technology Laboratory, Faculty of Animal Science, Universitas Andalas. Both strains were previously isolated from fermented fish (Budu) and molecularly identified by *16S rRNA* gene sequencing for *S. harbinensis* and internal transcribed spacer region sequencing for *P. kudriavzevii*, showing ≥99% sequence similarity to reference strains.

Stock cultures were preserved at −80°C in media supplemented with 20% (v/v) glycerol. Before experimentation, *S. harbinensis* was revived in de Man, Rogosa, and Sharpe broth and incubated anaerobically at 37°C for 24–48 h until the mid-log phase (OD_600_ ≈ 0.8). *P. kudriavzevii* was cultured in yeast peptone dextrose medium at 35°C–37°C for 24–48 h to reach the exponential phase.

Cell concentrations were adjusted to approximately 1 × 10¹² colony-forming units (CFU)/mL for each strain. The inoculum was prepared by mixing bacterial and yeast cultures at a 1:1 ratio by CFU count. The final inoculum dose used for incubation was 4% (v/v) of the total fermentation volume.

### Rumen fluid collection and *in vitro* incubation

Rumen fluid was collected immediately after slaughter from adult goats (approximately 45–55 kg body weight) fed a conventional diet consisting of forage and concentrate at a ratio of approximately 60:40. Rumen contents were collected within 15 min post-slaughter, filtered through four layers of sterile cheesecloth, and maintained under continuous CO_2_ flushing to ensure anaerobic conditions.

The rumen fluid pH at collection ranged from 6.5 to 6.8. The filtered rumen fluid was maintained at 39°C before incubation and used within 1 h of collection.

### Substrate composition and incubation medium

The incubation substrate consisted of 60% forage and 40% dry matter (DM) concentrate. The forage component primarily consisted of dried grass, whereas the concentrate comprised corn meal, soybean meal, rice bran, and mineral–vitamin premix.

The chemical composition of the substrate (% DM) was as follows: dry matter, 90.2%; crude protein, 14.8%; neutral detergent fiber, 42.5%; acid detergent fiber, 27.1%; ether extract, 4.2%; and ash, 8.1%. The substrates were dried at 60°C, ground to pass through a 1-mm sieve, and stored in airtight containers before use.

### Experimental design and treatment

A central composite design (CCD) within the response surface methodology (RSM) framework was used to evaluate the effects of three independent variables: C concentration (glucose; 0.1–0.3 g), N concentration (yeast extract; 5–15 g), and incubation time (24–72 h). A face-centered CCD consisting of 20 experimental runs, including six axial points and six replicates at the center point, was applied.

The response variables were individual SCFA concentrations (acetate, propionate, butyrate, iso-butyrate, valerate, and iso-valerate). A second-order polynomial model was fitted using Design-Expert software version 13 (Stat-Ease Inc., Minneapolis, MN, USA).

### *In vitro* incubation and postbiotic production

Each anaerobic fermentation unit contained 2.5 g of substrate, 200 mL of buffer solution (Tilley and Terry method), and 50 mL of rumen fluid. The prepared probiotic inoculum was added according to treatment specifications, followed by glucose and yeast extract as C and N sources, respectively.

Incubations were performed at 39°C under anaerobic conditions without agitation. At the end of the incubation period, microbial activity was halted by immersing the tubes in an ice-water bath.

### Sampling and SCFA analysis

Fermentation contents were centrifuged at 1,509 × *g* for 5 min at 4°C. Supernatants were collected and stored at −18°C until analysis. SCFA concentrations were determined using gas chromatography (GC; Shimadzu GC-2014, Japan) equipped with a flame ionization detector and a fused silica capillary column (30 m × 0.32 mm × 0.25 μm). Injector and detector temperatures were set at 250°C. The oven temperature was programmed from 100°C to 180°C at 10°C/min and held for 5 min. N was used as the carrier gas. SCFA standards were used for calibration, and results were expressed in millimolar units.

### Statistical analysis

Data were expressed as mean ± standard error of the mean. RSM modeling was performed using Design-Expert software version 13 (https://www.statease.com**, Stat-Ease, Inc., USA).** A one-way analysis of variance followed by Tukey’s post hoc test was conducted using SPSS version 29.0 (IBM Corp., NY, USA) to compare treatment means. Model adequacy was evaluated using R², adjusted R², predicted R², and lack-of-fit tests. Statistical significance was set at p < 0.05.

## RESULTS

### Model adequacy and statistical validation

The quadratic polynomial models generated for each SCFA were significant (p < 0.05). The coefficient of determination (R²) ranged from 0.82 to 0.94, indicating good agreement between the predicted and observed values. Adjusted R² and predicted R² values were in close agreement (<0.2 difference), confirming the model’s reliability. Lack-of-fit tests were non-significant (p > 0.05), demonstrating adequate model fitness. Residual plots showed a random distribution, supporting the validity of the model.

### Optimization of acetic acid production

The C concentration, N concentration, and incubation time had a significant influence on acetic acid productivity (Table 1). Among the tested C concentrations, 0.2 g yielded the highest productivity, with an average of 151.087 mM. In contrast, a lower C concentration (0.1 g) consistently produced lower productivity, while a 0.3 g C concentration, although yielding moderately high results, did not exceed the performance observed at 0.2 g. The optimal N concentration was 10 g, yielding the highest incubation output when combined with 0.2 g of C and 48 h of incubation. N levels of 5 g generally led to the lowest productivity, whereas 15 g yielded moderate but less consistent results. In comparison, incubation for 24 h led to moderate results, whereas treatments for 72 h showed relatively high productivity. The most favorable conditions for maximizing incubation productivity were identified as 0.2 g of C, 10 g of N, and 48 h of incubation.

**Table 1:** Optimization results for acetic acid production.

Run	Carbon concentration (g)	Nitrogen concentration (g)	Incubation time (h)	Average productivity (mM)
1	0.1	10	48	36.447
2	0.1	5	72	34.890
3	0.2	15	48	125.860
4	0.1	15	72	77.677
5	0.2	10	48	151.087
6	0.1	15	24	74.867
7	0.2	5	48	133.523
8	0.3	15	24	110.737
9	0.3	5	72	41.157
10	0.2	10	48	55.977
11	0.2	10	48	61.160
12	0.2	10	48	60.023
13	0.3	10	48	64.660
14	0.2	10	48	69.317
15	0.1	5	24	38.820
16	0.2	10	48	72.020
17	0.2	10	24	111.920
18	0.2	10	72	103.053
19	0.3	15	72	99.050
20	0.3	5	24	90.670

**Figure 1:**
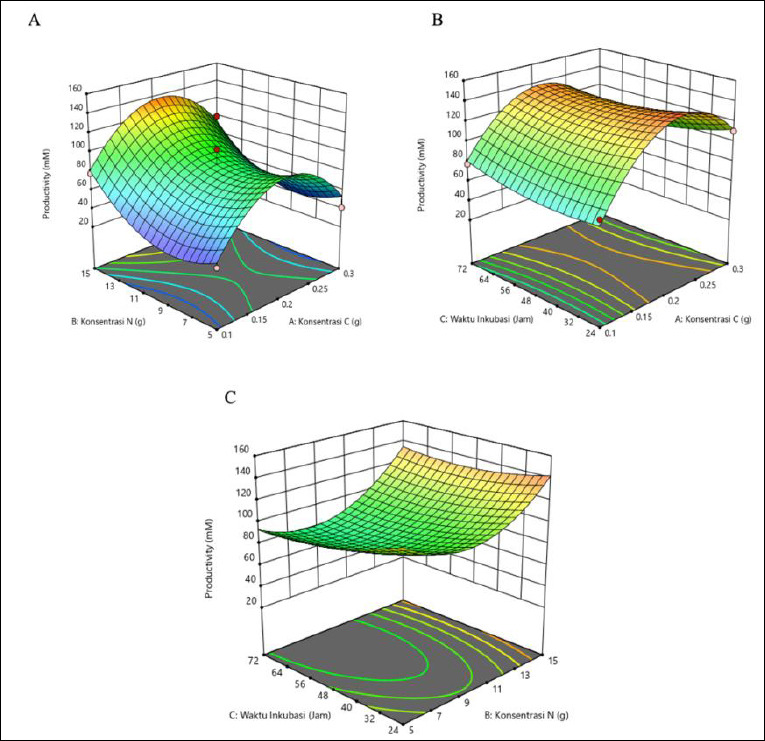
Response surface plots (3D) illustrating the effects of carbon (g), nitrogen (g), and incubation time (h) on acetic acid production (mM) by *Schleiferilactobacillus harbinensis* and *Pichia kudriavzevii*. The red regions indicate higher predicted responses, whereas the blue regions indicate lower responses based on the quadratic RSM model.

The response surface plots (Figure 1) in three-dimensional form showed color variations. The highest acetic acid production was indicated by the red area, corresponding to C concentration of 0.2 g, N concentration of 15 g, and incubation time of 24 h. The graph shows several regions in different colors, with the blue area indicating lower acetic acid productivity and the red area indicating higher productivity.

### Optimization of propionic acid production

The highest propionic acid productivity of 75.177 mM (Table 2) was observed at a low C concentration (0.1 g), a high N concentration (15 g), and a longer incubation time (72 h). N plays a crucial role, with higher concentrations generally correlated with increased productivity. Incubation time also affects productivity, with longer incubation times primarily enhancing output at favorable C and N concentrations.

**Table 2:** Optimization results for propionic acid production.

Run	C concentration (g)	N concentration (g)	Incubation time (h)	Average productivity (Mm)
1	0.1	10	48	27.190
2	0.1	5	72	34.467
3	0.2	15	48	29.763
4	0.1	15	72	75.177
5	0.2	10	48	22.843
6	0.1	15	24	63.697
7	0.2	5	48	22.147
8	0.3	15	24	16.770
9	0.3	5	72	15.583
10	0.2	10	48	36.517
11	0.2	10	48	31.167
12	0.2	10	48	39.850
13	0.3	10	48	34.420
14	0.2	10	48	19.707
15	0.1	5	24	12.923
16	0.2	10	48	54.633
17	0.2	10	24	32.140
18	0.2	10	72	42.130
19	0.3	15	72	47.327
20	0.3	5	24	22.957

### Optimization of butyric acid production

The results showed that butyric acid productivity was affected by C and N concentrations and incubation time (Table 3). Maximum productivity (17.443 mM) was recorded at 0.1 g of C, 15 g of N, and 24 h of incubation. Higher N concentration and shorter incubation time resulted in increased productivity, particularly when C was low. Conversely, lower productivity was associated with longer incubation times and low N or high C concentrations. These observations emphasize the importance of optimizing N levels and incubation duration to enhance butyric acid production. The response surface plots (Figure 3) support this result, where the green region rather than the red region represents the highest productivity, corresponding to the conditions of 0.1 g C, 15 g N, and 24 h of incubation.

**Table 3:** Optimization results for butyric acid production.

Run	C concentration (g)	N concentration (g)	Incubation time (h)	Average productivity (mM)
1	0.1	10	48	3.747
2	0.1	5	72	1.603
3	0.2	15	48	1.477
4	0.1	15	72	12.347
5	0.2	10	48	0.857
6	0.1	15	24	17.443
7	0.2	5	48	3.463
8	0.3	15	24	7.837
9	0.3	5	72	1.123
10	0.2	10	48	6.390
11	0.2	10	48	4.223
12	0.2	10	48	4.853
13	0.3	10	48	4.573
14	0.2	10	48	2.203
15	0.1	5	24	1.997
16	0.2	10	48	11.027
17	0.2	10	24	7.413
18	0.2	10	72	7.307
19	0.3	15	72	6.810
20	0.3	5	24	3.450

**Figure 2:**
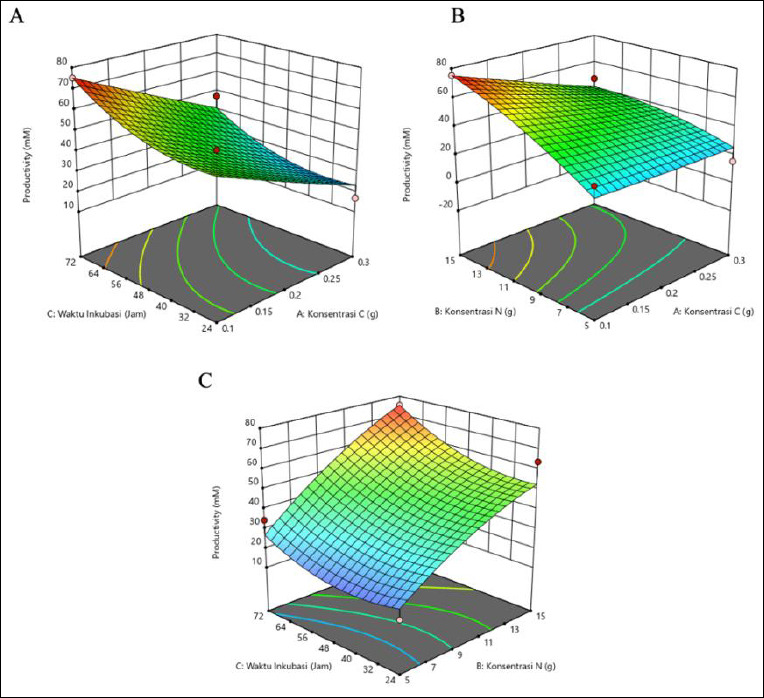
Three-dimensional (3D) response surface plots showing the interactive effects of carbon (C) concentration, nitrogen (N) concentration, and incubation time on propionic acid production (mM). Maximum production was predicted under low C (0.1 g), high N (15 g), and extended incubation (72 h) conditions.

**Figure 3:**
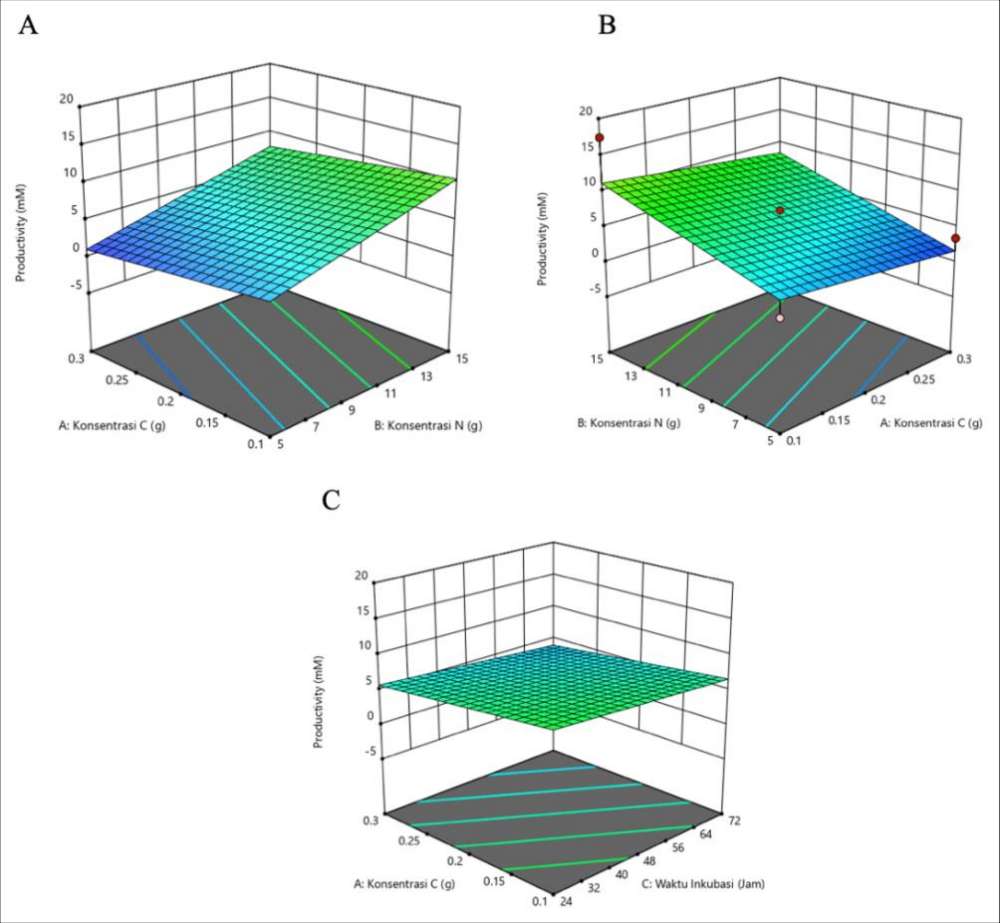
Response surface plots (3D) of butyric acid production (mM) by *Schleiferilactobacillus harbinensis* and *Pichia kudriavzevii* showing (A) carbon concentration, (B) nitrogen concentration, and (C) incubation time.

### Optimization of isobutyric acid production

**Figure 4:**
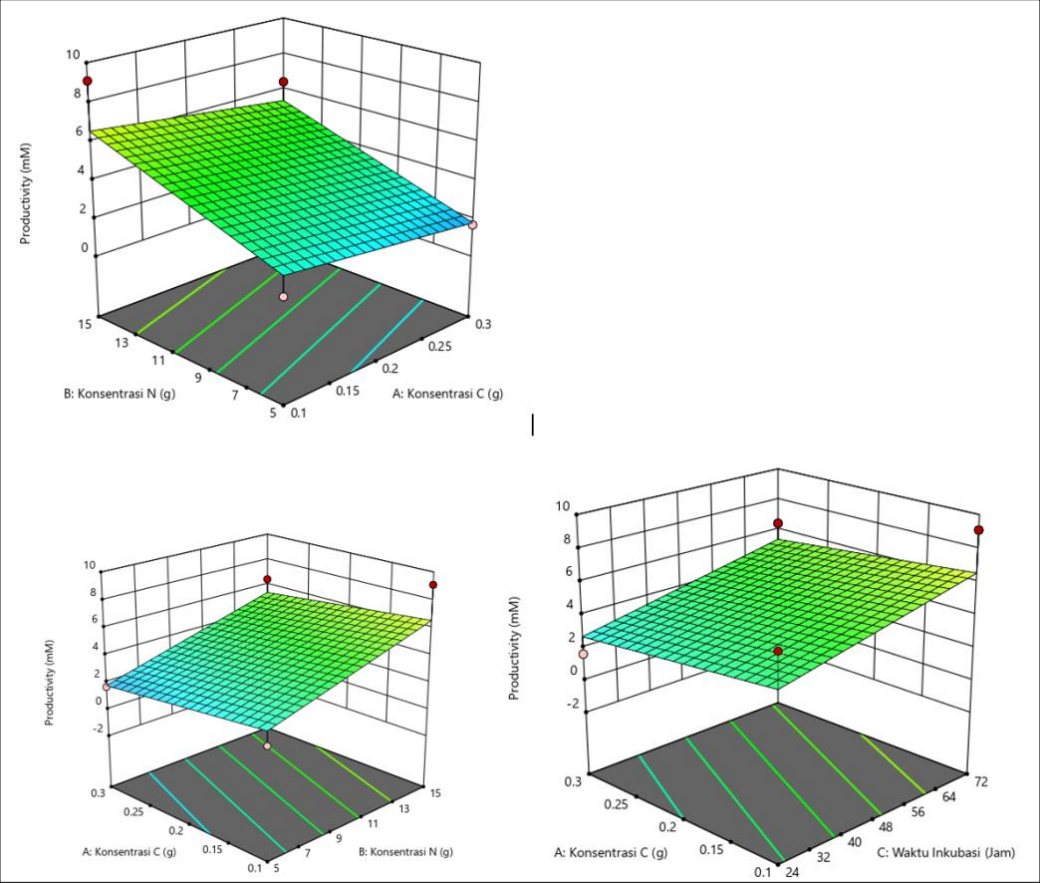
Response surface plots (3D) of isobutyric acid production (mM) by *Schleiferilactobacillus harbinensis* and *Pichia kudriavzevii* showing (A) carbon concentration, (B) nitrogen concentration, and (C) incubation time.

The productivity of isobutyric acid was affected by C and N concentrations and incubation time (Table 4). The highest productivity occurred at a high N concentration of 15 g and a longer incubation time of 72 h, with a low C concentration of 0.1 g. Moderate C (0.2 g) and 48-h incubation produced consistent results, whereas short incubation (24 h) generally led to lower productivity. The outcome depends on the interaction of all three factors. The response surface plots (Figure 4) show that the highest isobutyric acid production was indicated by the green area, corresponding to a C concentration of 0.1 g, a N concentration of 15 g, and an incubation time of 72 h.

**Table 4:** Optimization results for isobutyric acid production.

Run	C concentration (g)	N concentration (g)	Incubation time (h)	Average productivity (mM)
1	0.1	10	48	1.393
2	0.1	5	72	1.983
3	0.2	15	48	2.337
4	0.1	15	72	9.100
5	0.2	10	48	1.160
6	0.1	15	24	5.970
7	0.2	5	48	2.693
8	0.3	15	24	1.643
9	0.3	5	72	1.680
10	0.2	10	48	2.487
11	0.2	10	48	1.917
12	0.2	10	48	2.337
13	0.3	10	48	2.190
14	0.2	10	48	1.600
15	0.1	5	24	0.377
16	0.2	10	48	5.453
17	0.2	10	24	1.247
18	0.2	10	72	4.177
19	0.3	15	72	6.437
20	0.3	5	24	0.887

### Optimization of valeric acid production

**Figure 5:**
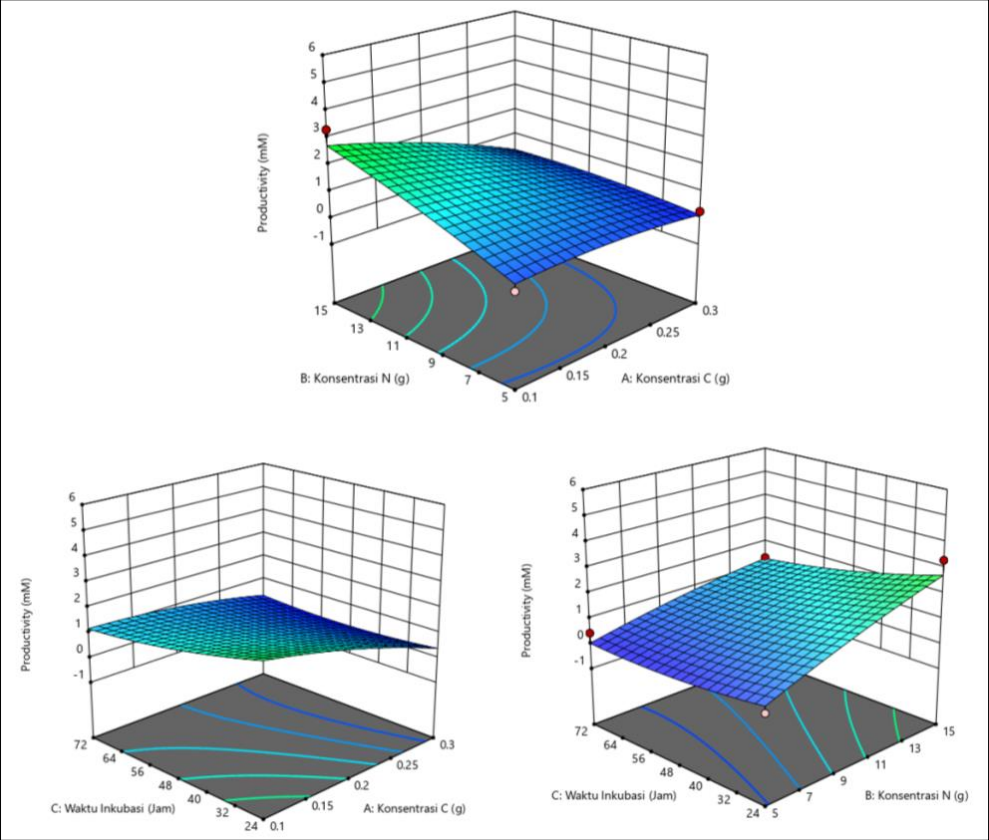
Response surface plots (3D) of valeric acid production (mM) by *Schleiferilactobacillus harbinensis* and *Pichia kudriavzevii* showing (A) carbon concentration, (B) nitrogen concentration, and (C) incubation time.

The average productivity varied with C and N concentrations and incubation time (Table 5). The highest productivity was observed at 0.1 g of C, 15 g of N, and 24 h (3.340 mM), followed by 0.2 g of C, 10 g of N, and 48 h (4.527 mM), suggesting that specific combinations are more effective. In general, a high N concentration (15 g) and shorter incubation time (24 h) produced better results. The interaction among all three factors played a crucial role in productivity optimization. The response surface plots (Figure 5) showed that the highest valeric acid production was indicated by the green area, corresponding to C concentration of 0.1 g, N concentration of 15 g, and incubation time of 24 h.

**Table 5:** Optimization results for valeric acid production.

Run	C concentration (g)	N concentration (g)	Incubation time (h)	Average productivity (mM)
1	0.1	10	48	0.243
2	0.1	5	72	0.367
3	0.2	15	48	0.360
4	0.1	15	72	1.253
5	0.2	10	48	0.133
6	0.1	15	24	3.340
7	0.2	5	48	0.940
8	0.3	15	24	0.570
9	0.3	5	72	0.240
10	0.2	10	48	0.437
11	0.2	10	48	0.050
12	0.2	10	48	0.150
13	0.3	10	48	0.783
14	0.2	10	48	0.770
15	0.1	5	24	0.147
16	0.2	10	48	4.527
17	0.2	10	24	0.930
18	0.2	10	72	0.527
19	0.3	15	72	0.513
20	0.3	5	24	0.243

### Optimization of iso-valeric acid production

**Figure 6:**
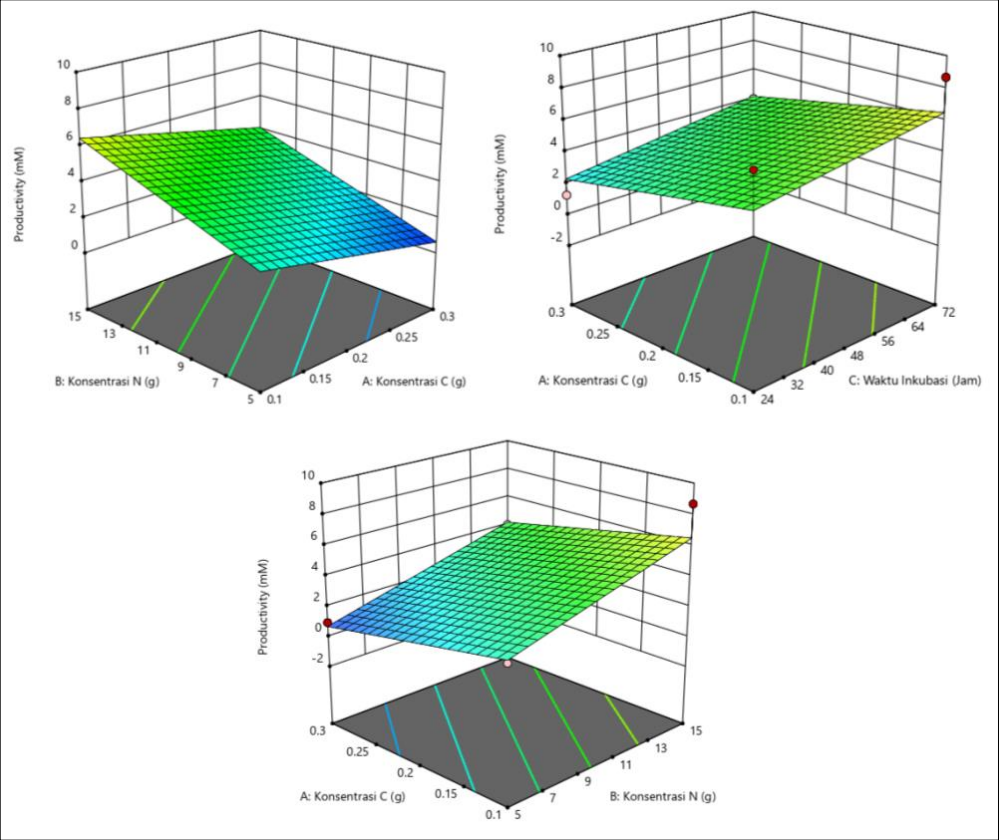
Response surface plots (3D) of iso-valeric acid production (mM) by *Schleiferilactobacillus harbinensis* and *Pichia kudriavzevii* showing (A) carbon concentration, (B) nitrogen concentration, and (C) incubation time.

Achieving maximum productivity depends on a specific combination of C and N concentrations and incubation duration (Table 6). The highest iso-valeric acid productivity was produced at a high N concentration (15 g), particularly when paired with either extended (72 h) or shortened (24 h) incubation times, yielding productivity of 8.690 mM and 6.903 mM, respectively. Consistently good results were also obtained with moderate C concentration (0.2 g) and 48 h of incubation. As illustrated in the response surface plots (Figure 6), the optimal production zone shown in green corresponds to a C concentration of 0.1 g, a N concentration of 15 g, and an incubation time of 72 h.

**Table 6:** Optimization results for iso-valeric acid production.

Run	C concentration (g)	N concentration (g)	Incubation time (h)	Average productivity (mM)
1	0.1	10	48	1.863
2	0.1	5	72	2.850
3	0.2	15	48	3.590
4	0.1	15	72	8.690
5	0.2	10	48	1.410
6	0.1	15	24	6.903
7	0.2	5	48	2.367
8	0.3	15	24	1.290
9	0.3	5	72	0.963
10	0.2	10	48	2.340
11	0.2	10	48	1.403
12	0.2	10	48	2.453
13	0.3	10	48	2.003
14	0.2	10	48	1.230
15	0.1	5	24	0.263
16	0.2	10	48	4.987
17	0.2	10	24	1.217
18	0.2	10	72	3.107
19	0.3	15	72	4.030
20	0.3	5	24	0.633

## DISCUSSION

### Novel metabolic interactions in bacterial–yeast co-culture systems

For the first time, this study demonstrates that a co-culture of *S. harbinensis* and *P. kudriavzevii* exhibits nutrient-dependent metabolic switching under rumen-simulated conditions. The yeast likely contributes to redox balance and precursor supply, while the LAB directs C flux toward SCFA synthesis. This synergistic interaction explains the enhanced production of propionate and branched-chain SCFAs in low C and N-rich environments.

### Interactive effects of C, N, and incubation time on SCFA production

Moderate C availability (0.2 g) favored acetate production, reflecting overflow metabolism under sufficient glycolytic flux. In contrast, C limitation combined with N enrichment redirected metabolism toward propionate, butyrate, and iso-SCFAs, likely through enhanced amino acid catabolism and Wood–Werkman and Ehrlich pathway activation. Extended incubation further intensified iso-SCFA formation, supporting their classification as secondary metabolites.

### Comparison with previous postbiotic fermentation studies

Most postbiotic optimization studies rely on monocultures or synthetic media. The present rumen-based co-culture system more accurately mimics host-associated fermentation and reveals metabolic dynamics that are not observed in conventional de Man, Rogosa and Sharpe and Yeast Peptone Dextrose systems systems. The findings extend previous reports by demonstrating that SCFA spectrum modulation can be controlled through nutrient management rather than strain replacement.

### Effect of glucose concentration on SCFA production

Glucose is a fundamental energy source for microbial incubation, driving glycolysis and leading to pyruvate production, a key precursor for the biosynthesis of SCFAs. Acetic acid production was highest at a glucose concentration of 0.2 g. A decline in acetate production occurred when the glucose level exceeded or fell below this concentration. As a primary substrate for *S. harbinensis* and *P. kudriavzevii*, C is essential to support acetogenic metabolism without triggering substrate inhibition or metabolic overflow. Excessive C input can lead to acidification of the medium, disrupting microbial balance and subsequently slowing down acetate biosynthesis [12].

In contrast to acetic acid, propionic acid reached the highest production at a glucose concentration of 0.1 g by *S. harbinensis* and *P. kudriavzevii*. Excess glucose can trigger acid stress and inhibit the growth of propionate-producing microbes such as *Propionibacterium* spp. [13]. Furthermore, glucose overload induces acid stress, resulting in the inhibition of key enzymes that play a role in the succinate–propionate pathway [14]. Low glucose availability may help establish a favorable redox environment for propionate synthesis under anaerobic conditions.

Butyric and isobutyric acid production by *S. harbinensis* and *P. kudriavzevii* was optimal at a low glucose concentration of 0.1 g. The limited C supply in the environment promotes more efficient energy conservation through butyrate incubation, as observed in *Clostridium* species [15]. Moreover, branched-chain fatty acids such as iso-butyrate, which are often derived from amino acid catabolism, are not severely dependent on high carbohydrate availability, further supporting the efficiency of low C conditions for production [16, 17].

In contrast to the production of other SCFAs, the production of valeric and iso-valeric acids by *S. harbinensis* and *P. kudriavzevii* demonstrated dual production peaks at C concentrations of 0.1 and 0.2 g. N levels and incubation time also influenced the peaks. This suggests that C availability is not the primary determining factor for longer-chain SCFAs, but the interaction between C, N, and incubation time plays a more critical role [18].

### Effect of N concentration (yeast extract) on SCFA production

The results showed that N source concentration, provided in the form of yeast extract, plays a significant role in the biosynthesis of SCFAs. Across all measured SCFAs, acetic, propionic, butyric, isobutyric, valeric, and iso-valeric acids, N availability had a significant effect on both acid production and formation time.

The highest productivity for most SCFAs by *S. harbinensis* and *P. kudriavzevii* was observed at a yeast extract concentration of 15 g/L, particularly when combined with low-to-moderate C concentration and optimized incubation times. Peak concentrations of propionic and iso-butyric acids were achieved under high N conditions (15 g/L) when paired with extended incubation (72 h) and a low C concentration (0.1 g). Similarly, butyric and valeric acids demonstrated higher productivity under the same high N conditions but with shorter incubation time (24 h), specifically at low C concentration.

The optimal N concentration for acetic acid production was 10 g, particularly when combined with 0.2 g of C and a 48-h incubation time. A lower N concentration (5 g) consistently resulted in the lowest productivity, whereas a higher level (15 g) produced moderate productivity. These results suggest that N functions as a regulatory factor in balancing cell growth and metabolite production. Excessive N may promote biomass accumulation at the expense of product formation, whereas insufficient N can limit enzymatic activity and reduce cell viability [19].

For propionic acid, a high N concentration (15 g) combined with low C (0.1 g) and an extended incubation time (24–72 h) resulted in the highest productivity (75.177 mM). This is in line with previous studies indicating that elevated N availability can enhance propionate biosynthesis by upregulating the expression of propionyl-CoA carboxylase and other key enzymes associated with the Wood–Werkman cycle [20, 21]. Based on the results, N-rich conditions may support prolonged metabolic activity and gradual product accumulation over time.

Branched-chain fatty acids, such as iso-butyrate and iso-valerate, are known to originate from the catabolism of branched-chain amino acids, including valine and leucine, and may benefit from the amino acid-rich composition of yeast extract [22]. Both acids exhibited peak productivity at an N concentration of 15 g, specifically under low C conditions and short to moderate incubation times. This suggests that high N levels support the metabolic vitality required for activation of butyrate synthesis pathways. Iso-butyrate, in particular, demonstrated a preference for longer incubation (72 h) under high N conditions, implying that N may also extend microbial activity and metabolite synthesis under controlled environments.

The production of valeric and iso-valeric acids increased under high N conditions (15 g). The highest valeric acid productivity (4.527 mM) was achieved at an N concentration of 10 g and an incubation time of 48 h, suggesting a slightly different optimal balance for this compound. However, under most other conditions, 15 g of N produced either higher or comparable results. Iso-valeric acid reached the peak productivity (8.690 mM) at 15 g N, particularly when combined with either 24 or 72 h incubation times. These results underscore the critical role of N in sustaining microbial metabolism across varying incubation times.

High N concentrations (particularly 15 g) generally promoted greater acid productivity across all acid types, although the degree of impact varied by acid type and incubation time. N is a key building block for amino acids, nucleic acids, and coenzymes, which are essential for cell growth and enzymatic functions. However, excess N can sometimes favor biomass over metabolite production, requiring a fine-tuned balance depending on the target product and microbial strain [23].

### Effect of incubation time on SCFA production dynamics

Incubation time is a factor that influences microbial growth, substrate utilization, and metabolite accumulation. The results showed that incubation time (24, 48, and 72 h) significantly affected the production of various organic acids, with each acid demonstrating a different optimal incubation time depending on the C and N concentrations.

The highest acetic acid productivity by *S. harbinensis* and *P. kudriavzevii* was obtained after 48 h incubation with 0.2 g C and 10 g N. A shorter incubation time (24 h) resulted in moderate productivity, while extending the time to 72 h did not lead to further improvement and, in some cases, even reduced productivity. This pattern is consistent with a previous study by Song *et al*. [24], who reported that maximum acetic acid accumulation occurred near the late exponential growth phase, when acetaldehyde dehydrogenase activity peaked before a drop in pH inhibited metabolism. The decline observed at 72 h can be attributed to substrate depletion or product inhibition effects common in prolonged incubation [25].

The production of propionic acid increased with extended incubation up to 72 h, particularly under low C (0.1 g) and high N (15 g) conditions. This suggests that *S. harbinensis* and *P. kudriavzevii* require prolonged metabolic activity to convert intermediates through the Wood–Werkman cycle. Zhang *et al*. [26] similarly reported that propionate productivity peaks during the early to late stationary phase (48–96 h), driven by **Nicotinamide Adenine Dinucleotide + hydrogen** accumulation necessary for acrylate reduction to propionate.

The maximum butyric acid production (17.443 mM) occurred at 24 h with 0.1 g C and 15 g N, whereas productivity decreased when incubation was extended to 72 h. This pattern indicates that *S. harbinensis* and *P. kudriavzevii* achieved optimal butyrate synthesis during the late exponential phase, after which butyrate kinase activity was inhibited by acid accumulation and pH decline. Similar results were reported by Esquivel-Elizondo *et al*. [27], who observed a significant decrease in butyrate levels due to a metabolic transition toward solventogenesis or gas production during extended incubation.

Isobutyric acid showed a different pattern, indicating maximum productivity at 72 h under low C (0.1 g) and high N (15 g) conditions. The delayed production suggests that isobutyric acid formation is a secondary metabolic pathway relying on valine-derived precursor accumulation. The biosynthesis of isobutyric acid and valeric acid from the catabolism of branched-chain amino acids in *Saccharomyces cerevisiae* through the Ehrlich pathway comprises three reaction steps: transamination, decarboxylation, and oxidation [28].

Valeric acid showed dual optima at 24 h (0.1 g C, 15 g N) and 48 h (0.2 g C, 10 g N). This indicates a strong interaction between incubation time and substrate composition, with valerate synthesis depending on the acetate and butyrate precursor balance. Factors such as feedstock characteristics, biocatalyst type, reactor configuration, initial pH, organic loading rate, temperature, and retention time significantly influence the productivity of volatile fatty acids [29].

Iso-valeric acid followed a similar trend to isobutyric acid, achieving the highest productivity at 72 h with high N (15 g), while moderate productivity was obtained at 24 and 48 h. The extended optimum corroborates the results of Thierry *et al*. [30], who stated that iso-valeric acid and α-ketoisocaproic acid were the main products of leucine catabolism, which intensified during prolonged incubation.

### Implications for postbiotic optimization and application

To the best of our knowledge, this is the first study to optimize postbiotic production using a rationally selected LAB–yeast consortium, revealing the synergistic metabolic conversions that enhance SCFA yields under controlled rumen *in vitro* conditions. Unlike previous studies that focused on single metabolites or single-source inocula, this study uniquely models and optimizes a full SCFA spectrum using a multifactor RSM approach, enabling the prediction of metabolite shifts under varying nutrient conditions.

Acetate is maximized at moderate C (0.2 g) and N (10 g). Propionate, butyrate, isobutyric, and iso-valeric acids require very low C (0.1 g) and high N (15 g). This demonstrates that nutrient-driven metabolic routing can alter pathways depending on environmental conditions. Ad originality: The study reveals, for the first time, distinct nutrient-dependent metabolic switching in a mixed-culture system, highlighting how substrate limitation and N enrichment drive different SCFA pathways selectively. This study reveals, for the first time, distinct nutrient-dependent metabolic switching in a mixed-culture system, highlighting how substrate limitation and N enrichment drive different SCFA pathways selectively. Combining LAB and yeast strains with complementary metabolic roles can significantly enhance SCFA biosynthesis, offering a new strategy for developing enriched postbiotic formulations. High N with prolonged incubation disproportionately enhances iso-SCFA synthesis. Branched-chain SCFA formation in co-culture systems suggests that N-rich environments promote amino acid catabolism through the Ehrlich pathway.

Higher propionate production, which is associated with improved energy efficiency in ruminants, and increased butyrate levels that support gut barrier integrity and exhibit anti-inflammatory effects, as well as balanced acetate production, are associated with improved energy efficiency in ruminants. This creates a scalable blueprint for cost-effective, nutrient-based optimization of postbiotics. This study proposes a resource-efficient approach for industrial postbiotic production with implications in animal feed, functional foods, and microbial fermentation technologies by identifying low C, high N conditions that maximize energy-dense SCFAs. The integration of rumen-derived microbial ecosystems with controlled nutrient optimization provides an innovative bioprocessing platform that mimics the dynamics of natural host fermentation, an approach that has not been previously applied in postbiotic research.

## CONCLUSION

This study demonstrated that a co-culture of *S. harbinensis* and *P. kudriavzevii* can be effectively optimized for postbiotic production under rumen-simulated *in vitro* conditions using a multifactor nutrient strategy. C concentration, N availability, and incubation time significantly influenced SCFA profiles, confirming that metabolite production in mixed microbial systems is strongly nutrient-dependent. Acetate production was maximized at moderate C (0.2 g) and N (10 g) with 48 h incubation, whereas propionate, butyrate, iso-butyrate, and iso-valerate were favored under low C (0.1 g) and high N (15 g), highlighting a clear nutrient-driven metabolic shift. Extended incubation preferentially enhanced branched-chain SCFA formation, indicating their secondary metabolic nature and dependence on amino acid catabolism.

From a practical perspective, these findings provide a scalable framework for nutrient-based optimization of postbiotic production. The ability to selectively modulate SCFA composition through controlled C–N ratios offers opportunities to design targeted postbiotic formulations for ruminant nutrition, functional feeds, and microbial fermentation industries. Increased propionate may improve energy utilization efficiency, whereas elevated butyrate supports gut barrier integrity and anti-inflammatory responses, making this approach relevant for both animal health and industrial bioprocess development.

A major strength of this study is the integration of a rationally selected LAB–yeast consortium with RSM in a rumen-derived fermentation system, enabling simultaneous modeling of multiple SCFAs rather than single-metabolite outcomes. This system more closely reflects host-associated microbial ecosystems compared with conventional monoculture or synthetic-medium studies.

However, several limitations should be acknowledged. The work was conducted under controlled *in vitro* conditions, which may not fully replicate the complexity of the rumen environment or in vivo host responses. Additionally, only one bacterial–yeast combination and a limited nutrient range were evaluated, and functional validation of the produced postbiotics in animal models was not performed.

Future studies should investigate broader microbial consortia, alternative C and N sources, and scale-up fermentation strategies, as well as validate biological efficacy in vivo to confirm health and productivity benefits. Mechanistic studies exploring metabolic flux and gene-level regulation would further clarify the pathways responsible for nutrient-dependent SCFA switching.

In conclusion, this study establishes that nutrient-controlled co-culture fermentation represents a promising and resource-efficient strategy for enhancing postbiotic production, providing a scientifically grounded platform for the development of next-generation functional microbial products.

## DATA AVAILABILITY

All primary data generated during this study are contained within the manuscript; however, additional supplementary materials are available from the corresponding author upon reasonable request

## AUTHORS’ CONTRIBUTIONS

YM, LA, and TJS: Conception and design of the study. YM, LA, SS, RD, LRA, and TAB: Preparation of materials, data collection, and data analysis. YM: Methodology. LRA and TAB: Formal analysis and investigation. LA, YM, and LA: First draft of the manuscript. YM, TJS, SS, RD, and LRA: Writing–review and editing. All authors have read and approved the final version of the manuscript.
